# Socioeconomic impact of breast cancer on young women in Ghana: A qualitative study

**DOI:** 10.1002/nop2.590

**Published:** 2020-10-06

**Authors:** Merri Iddrisu, Lydia Aziato, Lillian A. Ohene

**Affiliations:** ^1^ Department of Adult Health School of Nursing and Midwifery University of Ghana Accra Ghana; ^2^ Department of Community Health Nursing School of Nursing and Midwifery University of Ghana Accra Ghana

**Keywords:** breast cancer, Ghana, socioeconomic, stigma, young women

## Abstract

**Aim:**

This study was undertaken to discover the socioeconomic impact of breast cancer on young women in Ghana.

**Methods:**

A qualitative exploratory and descriptive design was used to recruit 12 young women from the University of Ghana hospital, 37 Military hospital and Ridge hospital. Individual interviews were conducted face to face and data transcribed verbatim and analysed using content analysis.

**Results:**

Three themes emerged: *perceptions and beliefs; economic concerns; and secrecy*. Participants perceived that breast cancer was a test of faith, a spiritual disease that is contagious and disgraceful. Mostly, participants stopped work to cater for themselves, and as a result, they encountered financial challenges. Their challenges were compounded with conscious efforts to keep diagnosis secret to avoid being stigmatized.

**Conclusion:**

Young women living with breast cancer need support physically, economically and socially from healthcare providers, their families and the society at large.

## 1 INTRODUCTION

Improvement in diagnosis and management strategies of breast cancer has reduced the number of deaths associated with breast cancer with increasing survivorship among women (Miller et al., 2016). Worldwide, breast cancer is the leading cause of cancer deaths in women with a 15% death rate globally (Globocan, 2019). The incidence of breast cancer among women in the United States in 2018 stood at 11% as against that of Sub‐Saharan Africa which is 22.4% and Ghana, 20.4% (Ferlay et al., 2019). Studies further show that the negative effects of breast cancer diagnosis and treatment affect young women physically, psychologically and socially (Lan, Jiang, Li, Sun, & Ma, 2020; Rana et al., 2017). The diagnosis of breast cancer at a younger age could be very frustrating with a sense of isolation, neglect and withdrawal, which could make young women 15–49 years (WHO, 2006) feel to have lost control over their lives. Studies indicate that young women having breast cancer have concerns different from that of older women (Chua, DeSantis, Teo, & Fingeret, 2015; Yfantis et al., 2017) with a lack of literature on young women with breast cancer in Ghana. This study explored the socioeconomic experiences of young women diagnosed with breast cancer in Ghana.

## 2 BACKGROUND

Young women living with breast cancer experience social isolation, withdrawal, and unendurable reactions from society due to erroneous beliefs society has about breast cancer (Al‐Azri et al., 2014; Taha, Al‐Qutob, Nyström, Wahlström, & Berggren, 2012). The diagnosis of breast cancer in women remains secretive due to stigmatization, isolation and bad comments people make (Chen, Liu, Li, & Su, 2017). These women even refuse to disclose their diagnoses to families and friends because of perceived stigma and the behaviour of society towards breast cancer patients (Mehrabi, Hajian, Simbar, Hoshyari, & Zayeri, 2016). There are patients who also selectively disclose to people who they think are trustworthy (Smith, Dawson‐Rose, Blanchard, Kools, & Butler, 2016). However, some do not disclose to families because they do not want them to get worried, overprotect them or have pity for them (Thornton et al., 2014). On the contrary, others have no problem disclosing their diagnosis because, to them, having breast cancer, is not a criminal offense (Mehrabi et al., 2016). In some cultures, when someone gets breast cancer, it means the person is bewitched or cursed and people shun the sufferer's company for fear of incurring the wrath of their ancestors (Muliira, Salas, & O'Brien, 2017).

Asobayire and Barley (2015) indicated that cultural beliefs, stigma and perceptions attached to breast cancer in northern Ghana limit women from openly seeking health care. Similarly, Jiwa, Ofori‐Atta, and Goh (2015) reported that breast cancer is perceived a taboo in some cultures in Ghana, and therefore, people who detect abnormalities in their breasts keep their symptoms for months before reporting to health facilities or first seek for healing in churches (Iddrisu, Aziato, & Dedey, 2019). Stigma is also reported by Licqurish et al. (2017), women affected with breast cancer refuse to mention it so that they keep the respect of their families. Additionally, some societies regard breast cancer as a taboo and ladies whose mothers suffer breast cancer find it difficult getting husbands in their local communities (Taha et al., 2012). Ignorantly, some cultures see breast cancer as a communicable disease, and therefore, sufferers are shunned; their clothing, bowls, cups and spoons separated from that of the larger family as a preventive measure (Inan, Gunusen, & Ustun, 2016; Licqurish et al., 2017).

Apart from the beliefs associated with breast cancer, financial constraints and long distances to health facilities have been identified as challenges to treatment and factors that cause patients to report late to health facilities (Kohler et al., 2017). Ekwueme and Trogdon (2016) posit that expensive breast cancer diagnostic investigations recommended during treatment leave patients with untoward economic hardship. Further evidence revealed that treatment puts a burden on both the patients and their families and hinder their quality of life (Grosse Frie et al., 2018). The economic burden of breast cancer in young women is higher because these women become less productive due to negative treatment effects which results in work absenteeism, have reduced work hours and pay higher bills to get medical attention and counselling (Ekwueme & Trogdon, 2016; Pisu, Azuero, Benz, McNees, & Meneses, 2017). There are cancer patients who stop working to take care of themselves while others change jobs to lesser demanding ones to minimize stress on themselves (Swanberg, Nichols, Ko, Tracy, & Vanderpool, 2017). According to Cardoso, Harbeck, Mertz, and Fenec, (2016), women look for less stressful jobs that pay less resulting in a decline in income. The financial burden associated with breast cancer care has been cited as some of the reasons why some cancer patients abandon treatment (Sanuade et al., 2018).

Generally, most women in Ghana work in the informal sector. The typical Ghanaian woman is considered as a homemaker who is basically supposed to care for children, do household chores and engage in some menial jobs to keep the home. The few minority women who find themselves in the corporate world averagely earn lower income. Consequently, young women in Ghana mostly lack the financial independence necessary to make treatment decisions when afflicted with a condition like breast cancer, and breast cancer treatment is not covered by the national health insurance scheme. The aim of this study was to explore the socioeconomic impact of breast cancer treatment and care on young women in Ghana. The findings will inform policy change to address unique socioeconomic challenges of young women undergoing treatment for breast cancer.

## 3 METHODS

### 3.1 Research design

An exploratory descriptive approach to qualitative research was used to explore and understand young women's socioeconomic experiences with breast cancer. Not much has been done on the topic, hence a qualitative exploratory and descriptive design were considered appropriate (Polit & Beck, 2013).

### 3.2 Participants and setting

We conducted the study in three different hospitals and interviewed young women between the ages of 15–49 years, diagnosed with breast cancer, and living in the Accra Metropolis. Inclusion Criteria were haven been diagnosed with breast cancer, undergone some form of treatment and could speak English and Twi. For exclusion criteria, newly diagnosed patients and acutely sick patients on admission were not recruited. Twelve participants were recruited using purposive and snowball sampling techniques. Initially, participants were carefully selected to meet the study's inclusion criteria; later, the snowball technique was used to recruit other participants. The secrecy behaviours associated with communicating ones' diagnosis necessitate the snowball technique for recruiting some of the participants.

### 3.3 Data collection method

Qualitative individual interviews were conducted with twelve research participants. A formal permission was sought from the three facilities where data were collected. Young women with breast cancer who fell within the inclusion criteria and received treatment at these health facilities were sampled. Potential participants were recruited and given information sheets which was well explained in simple language. By means of phone calls, the researchers had individual agreements with participants to arrange for interview sessions. A semi‐structured interview guide was designed to collect data that assisted the researchers to keep track of the research focus. Interviews were conducted face to face with each participant and audiotaped with participants' permission. Participants' responses where probed to allow them share freely their views on the phenomenon. The participants were interviewed at their convenience in English. Each interview took about 35–60 min and was recorded with participants' consent. Saturation was achieved by the tenth participants when no new information relevant to the study was obtained. Additional two interviews were done to validate data saturation.

### 3.4 Data analysis

Data were analysed using thematic analysis, and Miles and Huberman (1994) approach to data analysis was adopted. Data analysis was done alongside data collection. Data collected were first transcribed verbatim; then, all the researchers read through the data several times to have meaning to what participants narrated. The researchers examined the data for ideas, thoughts, and words that were similar and interesting, and coded them. Similar codes were grouped as subthemes and very related subthemes clustered to form themes.

## 4 FINDINGS

A total of twelve young women diagnosed with breast cancer, receiving treatment and care were involved in this study. Table 1 presents the sociodemographic characteristics of the participants who were between the ages of 32–45 years. The table outlines the ethnic background, occupation, marital status and level of educational attainment of the participants. The time of diagnosis expressed in months, types of current treatment and whether or not the participants was registered with health insurance were also presented. Three major themes, eight subthemes and sample codes are presented Table 2.

**TABLE 1:** Participants' sociodemographic characteristics

Identity (woman)	Age (year)	Tribe	Occupation	Marital status	No. of children	Level of education	Duration diagnosis (months)	Treatment received	Health insurance
1	33	Ashanti	Lecturer	Married	3	Tertiary	48	Mastectomy chemotherapy	No
2	45	Ewe	Trader	Married	4	S.H.S.	18	Mastectomy hormonal therapy	No
3	32	Ga	Engineer	Single	0	Tertiary	24	Mastectomy alternative therapy	Yes
4	32	Ewe	Pharmacist	Married	2	Tertiary	7	Mastectomy radiotherapy	Yes
5	32	Ashanti	Journalist	Single	1	Tertiary	24	Chemotherapy	Yes
6	28	Ga‐Adangme	Seamstress	Single	0	S.HS.	11	Chemotherapy hormonal therapy	Yes
7	40	Akuapem	Secretary	Single	1	Tertiary	8	Chemotherapy	No
8	33	Ewe	Hairdresser	Divorced	1	J.H.S.	24	Chemotherapy	Yes
9	41	Ashanti	Trader	Married	2	J.H.S.	9	Mastectomy chemotherapy hormonal therapy	No
10	44	Ga	Trader	Married	2	S.H.S	48	Chemotherapy	Yes
11	39	Ewe	Teacher	Married	1	Tertiary	18	Chemotherapy	Yes
12	32.	Akuapem	Teacher	Single	1	Tertiary	12	Mastectomy	Yes

**TABLE 2:** Themes, subthemes and codes

Themes	Subthemes	Sample codes	
Perceptions and beliefs	Perception and beliefs of participants on causes of breast cancer	Dangerous disease kills faster Cancer deadly Evil disease A test from God	Cancer a Disgraceful disease Breast being the Heart of women
	Perception and beliefs of family and friends on breast cancer	Cancer contagious Mother afraid Separated cutleries Eating alone Panties discarded	Feels sad A caring man The solution surgery Shortens life Information scary
Socioeconomic concerns	Work and employment concerns	Weakness Cannot work Leave without pay Stopped work All money finished	Building project halted Want new job Business down Chemo drugs
	Cost of treatment and financial challenges	No money for surgery Drugs not available Expensive No money for medications No money for hematinic	I have no money Could not go for surgery Insurance not working Suspended chemotherapy
Secrecy	Non‐disclosure of diagnosis	Will not tell anyone Difficult to tell Not interested Feels disgraced	Unconcerned friends Friends‐not calling Own problem Friends staring at you
	Selective disclosure	Will not spread it Family aware Spiritual father informed I trust them	Details not known My decision Not necessary Boyfriends informed
	Perceived stigma	Rumours Workplace stigma Compound house She is sick Rejection	Money sacrifice Will not come close Should be secret Tell friends
	Isolation and withdrawal	Friends Unconcerned Always indoors Stays home Changed appearance	Staring at you Not more calls Cannot stand it Relocated No more friends

### 4.1 Perceptions and beliefs

This theme explains the participants' reported perceptions and beliefs about breast cancer. These perceptions and beliefs affected some participants' acceptance to comply with medical orders. Others believed that breast cancer was a test of their faith, and few believed that breast cancer was contagious. Two subthemes emerged: *perception of participants on breast cancer* and *perception of family and friends on breast cancer*.

#### 4.1.1 Perception of participants on breast cancer

Many participants had strong religious beliefs about the causes of cancer and cancer treatments. Consequently, it was revealed that some participants spent time in prayer camps praying for healing. The common perceptions and beliefs held by participants about breast cancer included; breast cancer being a disgraceful disease, caused by spiritual and evil forces or human enemies.

If it is not spiritual then why is that it was never detected from all the investigations I did and now confirmed when I cannot walk. Others too might be interested in your job position and once you are no more, they can occupy it like I am at home now. (W11)

Some also believed that the breast of every woman is precious and that breast cancer is disgraceful, deadly and dangerous.

…all that I know is that we the women our breast is our heart and once they temper with the breast you will not live long. In the beginning, I refused the surgery. (W10)

Some participants perceived that breast cancer is; “deadly, disgraceful, dangerous and a journey but not a destiny” to the sufferer. “…breast cancer is a very dangerous disease because if you joke with it, it will kill you just like that” (W5)

Once people get to know that you have cancer, they shout hey! Because everything that has to do with cancer is deadly so why should people get closer to you. (W6)

As for breast cancer, my sister, it's a disgraceful disease. There is nowhere I have not been to just to get healing but look at me here. (W8)

Most of the participants narrated that when one gets breast cancer, the person needs love and affection from family and loved ones to survive it. Others believed that an untimely death may occur if love ones, especially partners do not show love and support to breast cancer patients.

…You need love and affection from loved ones and if such is not given, it can kill you earlier than the disease itself. … (W8)

It is all about having a caring man around you who will go through thick and thin with you. (W7)

Due to some of the beliefs and perceptions participants had about breast cancer, two participants refused to go for surgery after diagnosis. They shared the belief that the breast is the heart of every woman and that once your breast is removed, you will die.

…Recently a lady died in our area and I heard she had breast cancer and went to do surgery. One lady said that the surgery is what killed her. (W9)

#### 4.1.2 Perception of family and friends on breast cancer

According to the participants, the perceived spiritual causes of cancer were reflective in the behaviours and attitudes of friends and families in daily interactions with such people.

A participant reported that her mother believed breast cancer is a contagious disease and therefore prevented her from feeding her only child. She also said that due to her disease state, she was not allowed to share plates and cutleries with the rest of the family. Instead, she was given her separate sets of kitchen wares

…my mum believes the disease can be transmitted so she does not allow me to eat with my son. I have separate bowls, spoon, and cup from that of the family. Even when I'm eating and my child comes and I feed him, my mother will take the boy away and will start talking. When I was admitted and I gave my panties to her to wash for me, she threw them away … I knew she was afraid she could be infected. I am sad she is doing that. (W5)

… my mother and siblings hate me and do not talk to me. I believe they can do anything to harm me. ……my auntie too envies me because I have my own house, so I believe my condition is spiritual and I realized it is coming from my mother's side. (W10)

### 4.2 Socioeconomic concerns

This theme has two subthemes which illuminate the employment difficulties and financial challenges participants encountered. Most of the participants reported that they stopped work to concentrate on their health. One participant said she took a year leave from work without pay. Almost all participants complained of huge hospital bills, and also, most of the treatments were not covered by the national health insurance scheme. Due to the financial challenges, one participant could not start the chemotherapy prescribed for her after surgery. Another participant struggled financially to complete subsequent sections of the chemotherapy.

#### 4.2.1 Work and employment concerns

Most of the participants abandoned their work to take care of themselves. Some participants cited treatment effects and stress as reasons for their inability to work.

I get stressed up and stress is not good so I had to slow down for a while. I finally withdrew from work totally. (W1)

My condition has affected my work a lot, my work is a practically oriented one and I have to go up and down with my students to see whether what I asked them to do they are doing it. But now I cannot move around. I feel dissatisfied and uncomfortable with my work output. (W11)

Some participants took leave from work without salary to recover before going back to work.

…I have asked for leave without pay for a year to recover fully. (W4)

…I have stopped work until I am done with chemo and my surgery. I do not have the strength to go to the market. (W9)

#### 4.2.2 Cost of treatment and financial challenges

Most of the participants complained of financial constraints and the high cost of bills with the treatment of breast cancer. The inability of the national health insurance to cover the full cost of treatment made it difficult for some participants to initiate treatment on time.

… because of financial challenges, I could not go for chemotherapy. I also realized that it is not just once but involves six cycles so there was no way I could afford it. More so, I was told insurance does not cover the drugs and they are expensive too. (W12)

I was told to pay three thousand cedis (about 500 dollars) for the surgery alone and I did not have money so I left…The laboratory investigations alone cost about six hundred ($) not to talk about the medications. (W2)

A participant lamented that she could not finance her surgery because she had spent all her monies taking care of herself. She is unable to complain when in distress because the doctors would not care about her financial status but would prescribe as she complains.

I was told if I do not go for the surgery, cancer can kill me but I do not have money to go for the surgery… I have spent all my money on this disease. Now getting the subsequent cycles of the chemo is also difficult. As for the doctors, they do not care whether you have the money or not. Once you complain they will prescribe so if something is worrying me, I am not able to complain again. (W8)

### 4.3 Secrecy

Subthemes under this theme include non‐disclosure of diagnosis, selective disclosure, perceived stigma, withdrawal and isolation,

#### 4.3.1 Non‐disclosure of diagnosis

Most of the participants preferred keeping their diagnosis to themselves because they did not trust people. A participant indicated that she could not tell her Pastor for the fear that the Pastor might use her diagnosis to preach from the pulpit.

I will not even tell my pastor because you cannot trust anyone… if I tell the Pastor and he uses it to preach from the pulpit what will I do, had I known is always, at last, my sister, I don't want to involve anybody in my affairs. (W12)

I do not want my colleagues at work to know that I have breast cancer. I am strong and I just want to keep my condition to myself and not even disclose to my closest friend. You know we ladies talk a lot. (W5)

#### 4.3.2 Selective disclosure

Some participants selectively informed their partners, children, close family members and a few significant others. Those who disclosed it to their partners and children did that because they trusted them.

It is only my husband who is aware I have breast cancer. (W9)

Nobody knows about it except a cousin of mine because when I went for the surgery, she came in at some point to help take care of my kids. My husband and my two kids are also aware, I opened up to them. (W10)

I have told almost all my family members about my condition except one of my old ladies who is not feeling fine. (W11)

Other participants informed those who were close, trustworthy and could keep a secret. In some cases, the disclosure was partial. According to some participants, the word cancer was not mentioned in their disclosure; however, the close relations only knew they were not well.

My siblings are aware of it but for my prayer group members it was my pastor

who told them that I have a tumour but they don't know the details? (W3)

My mother, my spiritual father and my boss at work are aware I have this disease. (W5)

Some participants did not disclose their breast cancer diagnosis because they perceived they may be stigmatized.

#### 4.3.3 Perceived stigma

The embarrassment or disgrace attached to breast cancer prevented participants from disclosing their diagnosis to some family members and significant others because they perceived they may be stigmatized and isolated.

I stay in a compound house with other families and if they get to know of my condition, they will not come close to me and they will spread it in the neighbourhoods. (W12)

Why should anybody know that I have breast cancer, they will also add their own story and spread it. It will become like a stigma on me and people will not get close to me anymore. (W7)

Participants believed that apart from the falsified speculations which might spread about their disease state, they also fear that people may lay blame and false accusations on them. For example, a participant said that people could accuse her of sacrificing her body parts like the breast for money rituals.

I stopped going for prayer sessions…, I have not told anyone because they can say I have used my breast for money rituals. You know, rumours spread like wildfire in our area and I cannot be their chewing stick so I decided to keep it a secret because I cannot tolerate it. (W9)

I have not told anybody because they will stigmatize me like I am a witch. They will talk as though I have used my breast for money and all sorts of some rituals. (W10)

Some participants also felt that if they share their diagnosis with people, especially their friends, they would associate every subsequent complaint to breast cancer. One participant believed that she would lose her respect, royalty and be blamed for bringing a bad disease into her Royal family.

…at the workplace, it also happens that if anything petty happens to you, they will pass comments like: ‘Oh this person she is sick don't mind her. (W3)

I come from a royal family. Among my siblings, I have a good marriage. I would not want to lose the respect people have for us with this deadly disease. (W9)

Due to perceived stigma, a participant did not seek support which is affecting the education of her children.

If I bring any maid to the house, she might tell people about my condition and I will be stigmatized. …At times I hear people ask why my last born is not going to school and he tells them mummy says I should not go today but the reason cannot be shared. (W9)

#### 4.3.4 Isolation and withdrawal

Some of the participants narrated that body changes in breast cancer treatment is obvious. To avoid being noticed of the body changes, they relocated to a new neighbourhood.

I have changed physically and if I continue to stay in the neighbourhood, people will begin to ask questions and I cannot stand it. … when my skin regains its colour, my hair grows and other things reverse after treatment, I will go back but for now, no! I am very sociable and cannot confine myself to the room so I think what I did is the best. (W7)

Other participants who did not relocate also withdrew from social programmes like wedding ceremonies, funerals, church service and other family programmes. They did so either because of their wounds after surgery, too weak and unable to walk or wheelchair‐bound due to cancer metastasis to the spine.

… I don't go to programmes any longer because of the wound. Now I don't go for weddings, funerals and other social functions because the wound is not healed for me to replace the breast with something. (W3)

… How can I go out if I'm not walking? My family is prepared to send me to programmes but I declined because when we get there… and people see me in the wheelchair, it is embarrassing. (W11)

Other participants did not attend programmes to avoid stressing themselves and exposure to infections because their immune system was compromised as a result of chemotherapy.

… my doctors said if you are doing chemo you should avoid stressing yourself and avoid public places so that you don't get infections. I know my immune system is down so I don't go for programmes at all. (W5)

## 5 DISCUSSION

This study's findings support previous studies that the socioeconomic experiences of young women diagnosed with Breast Cancer are challenging irrespective of race or status. For example, the social disparities in aetiological knowledge of cancers are well documented (McCutchan, Wood, Edwards, Richards, & Brain, 2015; Nguyen et al., 2016; Vijayasiri et al., 2018). Many of these studies recorded low knowledge among populations on cancer causes, prevention and treatments even among cancer patients (Ryan et al., 2015). Evidence shows that beliefs about the causes of breast cancer have high tendencies to influence acceptance and participation in cancer screening and treatment programmes (Khan, Leong, Ming, & Khan, 2015; Tranberg et al., 2016). Unsurprisingly, the inaccurate beliefs shared in this current study had an impact on participants' decisions to keep their diagnosis secret from families and friends. Negative beliefs such as breast cancer being a shameful disease from evil spirits and also contagious are suggestive of the social stigmatization of patients (Sanuade et al., 2018; Taha et al., 2012). In places where such beliefs are absent, cancer patients freely disclose their diagnosis (Meacham, Orem, Nakigudde, Zujewski, & Rao, 2016; Simon, Tom, & Dong, 2017).

The high cost of breast cancer treatments as reported in the study supports several similar findings from different context including low‐ and middle‐income countries (Ekwueme & Trogdon, 2016; Pisu et al., 2017). Currently, financing cancer treatment in Ghana comes as the sole responsibility of the individual patients and the treatment begins with a series of diagnostic investigations that are typically financed out of pocket payment (Twahir et al., 2019; Vanderpuye et al., 2017). Furthermore, the three available treatment strategies: chemotherapy, surgical interventions and radiotherapy are associated with exorbitant costs over a long period (Vanderpuye et al., 2017). The average cost of chemotherapy in Ghana ranges between $ 270–$ 360 depending on the facility one attends. Surgeries like removal of a lump or a breast may cost as high as $100–$200 (Giordano et al., 2016). In most cases, treating the side effects also come with additional costs all borne by patients as out of pocket payments (Palesh et al., 2018).

Culturally in Africa, sick people are expected to be shown sympathy by the family and society but in instances where the sickness has social and spiritual connotations, people hide their sicknesses to ward off social sympathy, prevent stigmatization and social isolation. This is predominant in Africa as reported by Muliira et al. (2017), in their integrative review on studies done in Africa between 2005–2015. The findings from their review revealed that women diagnosed with breast cancer are considered to have been bewitched and their husbands perceived them not whole. This could impact negatively on the well‐being of these women, and therefore, healthcare practitioners need to put in pragmatic informative strategies to correct societal beliefs and public perceptions about breast cancer (Asobayire & Barley, 2015).

It was realized that disclosure was not done because society, especially friends, would associate any ill‐health with their condition and would not receive attention as expected. These findings support that of Al‐Azri et al. (2014) with Omani women reported to unwillingly disclose their diagnosis to friends to keep friendship relations and maintain resilience. This is not surprising when one participant believed that she might lose respect and be blamed for bringing a bad disease into her royal family and refused to tell her family about her diagnosis. Equally, Licqurish et al. (2017) posit that women diagnosed with breast cancer refuse to tell others for fear of losing their families' respect. In a way to keep away from public ridicule, participants reduced their social interaction by abstaining from social gathering and some relocated. This is a common practice not only among breast cancer patients but also reported by Smith et al. (2016) where people diagnosed with HIV/AIDS relocated when their families and friends got to know of their health condition, some even stopped going to church to avoid being stigmatised through preaching. In the current study also, one person did not tell her pastor about her diagnosis for fear of the pastor using it for a sermon.

The economic burden of breast cancer in young women is higher than any other cancer type because young women diagnosed with breast cancer become less productive due to negative effects of treatment, they take days off from work to access health care, have reduced working hours and pay higher bills for medical attention and counselling (Ekwueme & Trogdon, 2016; Pisu et al., 2017). Correspondingly, this study found that the participants were all working before their diagnosis but due to the effects of treatment, most of them halted work for some time, abandoned work to care for themselves and others changed jobs to less demanding ones due to stress. Cardoso et al. (2016) support these current findings with their study result which indicated that women diagnosed with breast cancer change work for less demanding ones with lesser salary and less stress which results in income decline. Although some of them remained with their employers, they experienced dissatisfaction in their jobs for not being able to put up their best (Tamura, Sakaguchi, & Yamanaka, 2019).

The high cost of treatment gave participants financial challenges. The national health insurance of Ghana does not cater fully for the cost of treating breast cancer. Participants paid for their laboratory investigations and treatment cost, but this was not the case in the advanced countries. Williams and Jeanetta (2016) reported that health insurance covered for the full treatment cost of breast cancer for most patients in their study. With these financial challenges faced by these women, they had to rely on family members, employers and other people for assistance to enable them go through treatment. A lot of studies have reported similar findings (Sanuade et al., 2018; Twahir et al., 2019; White‐Means, Dapremont, Davis, & Thompson, 2020). Amid the financial difficulties, some employers promised to assist some participants with payment of hospital bills; however, these promises are often informally arranged and rarely redeemed.

## 6 CONCLUSION

Breast cancer in young women comes with a lot of social and economic challenges. The disease puts a lot of financial burden on young women when they are unable to work as a result of negative treatment effects. Society's deleterious beliefs and perceptions about breast cancer make these women perceive they are stigmatized and therefore isolate themselves.

## 7 RELEVANCE TO CLINICAL PRACTICE

There is a need for healthcare providers to care for breast cancer patients in way that would not communicate further stigmatization within the health facilities. Again, healthcare providers should intensify public education on the risk factors of breast cancer and also demystify the numerous perceptions and beliefs about the disease.

## CONFLICT OF INTEREST

No conflict of interest exists.

## AUTHOR CONTRIBUTIONS

MI: Conceptualization of the study. She is responsible for the selection of the methodology, investigation, resource mobilization and data analysis. She participated in the initial drafting and editing of the manuscript. LAO and LA: Conceptualization, selection of the methodology, supervision and formal analysis of the study. They were also involved in the drafting, reviewing and editing of the manuscript.

## ETHICAL APPROVAL

Ethical approval was gained from the Noguchi Memorial Institute for Medical Research, Ghana, NMIMR‐IRB CPN 111/15‐16.

## Data Availability

The data sets for this study are available from the corresponding author on reasonable request.
